# The Thyroid Gland

**Published:** 1915-11

**Authors:** W. L. Crosthwait

**Affiliations:** Waco, Texas


### The Thyroid Gland.

BY W. L. CROSTHWAIT, M. D., WACO, TEXAS.

At this time our ideas of the physiological functions, pathological changes and surgical importance of all of the ductless glands are undergoing revision and enlargement. The thyroid being one of the most important of the group has come in for its share of consideration. When we come to study the thyroid from a pathological standpoint and to deal with it either therapeutically or surgically we arrive at five basic and fundamental propositions, towit:

1. The thyroid is essential to the growth and health of the individual, its functional activity corresponding to the physiological life of the ductless glandular system, of which it is the most integrant and reciprocal part.

2. Hyperactivity or hypoactivity of the thyroid may be counteracted or compensated for by the eliminative or reciprocal organs within certain bounds, varying with different individuals, beyond which limits such over or under activity will result in such disturbance of nervous, nutritional and metabolic equilibrium as to cause characteristic symptoms and pathology.

3. The thyroid secretion is distributed through both the vascular and lymphatic systems, an excess of secretion if taken up and not properly neutralized is productive of a systemic toxemia, which with all of its attending pathology and symptoms is best designated *hyperthyroidism*.

4. A deficiency of thyroid secretion is productive of certain changes in metabolism, due to some cause not positively known, which together with its characteristic pathology and symptoms is designated *hypothyroidism*.

5. The hyperactivity of the thyroid is due to some stimulus endogenous in character and resulting from cellular changes in the gland itself in the process of reversion back to some former function.

If these facts are accepted, the conclusion is that goiter, the common name for enlarged thyroid glands, must be treated by directing attention to the gland itself with the object of reduction of the hypersecretion within the bounds of normal health and the final conclusion is that in most cases it is a surgical disease.

We will now take up each of the five phases of this subject separately. A clear comprehension of the first proposition must be based upon a full knowledge of the embryology, normal histology, physiological function and anatomy of the thyroid gland.

The thyroid gland in common with the respiratory organs develops from the pharyngeal entoderm. It is a compound tubular gland, and in the early part of its development it is connected with the mucous surface through the thyroglossal duct, which opens in the foramen cecum, which duct undergoes obliteration before the gland attains its full development in embryo, thus converting it into a ductless gland. The fully developed thyroid is composed of tubular or closed acini, which are lined with single layers of columnar epithelium. The tubules are united by areolar tissue into lobules which lobules are in turn bound together by connective tissues constituting lobes. The lobes are enclosed in a fibrous envelop, thus constituting the gland. This is nature's wise arrangement to preserve a part of the gland in its functional activity when one or more lobes are affected.

The cell secretion is characteristic colloid; that is, the residue is colloid or a viscid yellowish substance which fills the acini.

The alveolar spaces between the lobules and lobes contain broken up epithelium, leucocytes, migrated plasma and red blood cells. All of this make up the bulk of the enlargement in all colloid goiters, and constitutes a considerable portion of all enlarged glands.

The thyroid gland begins its development early in fetal life, and attains its full development during the active period of adult life, and atrophies in old age. Its greatest functional activity corresponds to the active sexual life of the individual.

Some writers or rather investigators believe the thyroid was formerly a sex organ. This theory is strengthened by observation of the changes which take place in the gland during adolescence, the menstrual period, the honeymoon and pregnancy.

If we assume that the cellular activity of the thyroid is governed by the same laws of metabolism as characterize the other glands of the body, in that the cells have the inherent power of selecting and assimilating tissue or substances as needed for nutrition and functional activity, and that they have the power to change such substances as are thus taken up and converting them into new substances which are delivered to reciprocal organs, we are forced to conclude that the thyroid performs a function quite necessary to the maintenance of normal health.

Our second conclusion is based upon the above assumption; in other words, that the thyroid normally elaborates a secretion, the distribution and absorption of which is essential to maintain metabolic equilibrium, and that in the said production and distribution of said secretion that it is governed by the same or similar reciprocal or compensatory laws as govern other secreting glandular organs of the body. Therefore, from analogy, we reason that individuals will vary greatly in their ability to handle or neutralize an excess of secretion. This also varies at different ages and under different conditions of health and environment of the individual. This must be true, for we often see persons with well marked cases of hyperthyroidism outliving the disease, and we sometimes see exophthalmic goiters reverting back to simple ones. Whatever virtue there may be in dietetic or climatic conditions is due to this fact. However, it must be remembered that there is a relatively wide variation in all simple goiters as to their elaboration of secretion which is well within the range of approximate normal health. Also such hyposecretion or hypersecretion may manifest itself occasionally when theoretically it should constantly be present.

We therefore conclude that the eliminative and reciprocal organs may be able to maintain approximately the ratio to the other secretions for some time during either under or over activity of the thyroid. But it must also be borne in mind that the break in the equilibrium of the ductless glands may occur suddenly, due to some acute illness or to shock. This is a very interesting phenomenon, and is responsible for the acute exacerbations we often notice in goiter patients, and also responsible for the hyperthyroidism following operations for the disease, and the writer does not doubt that it is responsible for the tetany and other symptoms of hypothyroidism which sometimes follow thyroid surgery.

From what we have said it is apparent that a diagnosis of hyperthyroidism or hypothyroidism may not be made early, and, in fact, not until the perverted thyroid function has seriously impaired the health of the patient, and many times not until permanent lesions have been produced in the heart, kidneys, etc. This should impress upon us the importance of a close study of inaugural symptoms.

We come now to the third proposition. The thyroid gland is very vascular. Its veins and arteries are unusually large and freely anastomose. It is said that all the blood in the body passes through the thyroid every two hours. The gland is also plentifully supplied with lymphatics. The deeper lymph vessels begin as spaces between the bundles of fibrous tissue close to the aciniform tubules, the superficial occupying the septa between the lobules. The deeper and the superficial unite to form the trunk lines which carry off the secretion.

The secretion of the thyroid is taken up slowly when normal conditions prevail, and when the gland becomes hyperactive the absorption must be more rapid, and this process must increase directly in proportion to the increased functional activity of the gland until the final break comes.

The size of the gland is not always a fair index as to the amount of thyroid secretion elaborated. In some cases there will be great cellular activity with very little enlargement; on the other hand, there will be very great enlargement with very little or no excess secretion, and in some cases with under secretion. The reason for this was pointed out in discussing the histology of the gland. We would suggest, however, that an excess of secretion may be held in the gland owing to the blocking of the lymphatics, in which cases the colloid material may have the power of holding the secretion, which retention must result in enlargement of the gland by increased deposit of colloid with idiothyroglobin until cell activity in large areas of the gland is destroyed. This is the probable cause of remission of symptoms in some goiters and also the theoretical causes of the reversion of exophthalmic goiters back to simple ones.

Our fourth conclusion, viz., the deficiency of functional activity of the gland, needs no further discussion. Therefore, passing to our final conclusion, however, we will speak briefly of the etiology of goiter.

The hyperactivity of the thyroid is due to some stimulus in the form of a toxin. Formerly it was thought that goiter was caused by some climatic conditions. Certain localities, such as the Rocky Mountain district and the Swiss Alps, have long been noticeable for the large number of goiters among both the native and adopted population. For a long time it was believed that some element in the water had something to do with the enlargement or overdevelopment of the thyroid.

All recent observers believe that the real cause of extra cellular activity, enlargement and oversecretion is due directly to a toxin. This toxin is undoubtedly endogenous. The only question which we are in doubt is as to whether it is a product of some peculiar activity of the gland itself or whether it is due to deficiencies of other reciprocating parts of the glandular system. Observation upon the results of ligation of the blood supply and partial removal of the gland would lead us to think that the toxin is a product of the gland itself.

To speculate upon just what this particular toxin may be or where it might originate or as to how it acts to stimulate the gland to overactivity is of no great value in connection with the surgery of goiter other than the assurance which is afforded that if the diseased gland is removed the primary cause which gave rise to its pathology will disappear and no further phenomena will be observed other than that due to impairment of the system resulting directly from the goiter.

It is obvious from what we have said that we believe that the treatment of goiter must be directed to the gland itself. We believe that most goiters are amenable only to surgical treatment.

The operative treatment of diseases of the thyroid has come to occupy an invulnerable place in the field of surgery. Through the brilliant work of surgeons of world-wide renown, who count their cases by the thousands, aided by the internist and pathologist, the indispensable coworkers of the surgeon, the technic and indications for the various operative procedures, such as ligation of the inferior or superior blood-vessels, complete or partial extirpation, etc., or enucleation, have been placed upon a rational basis and so well established that surgeons of less experience may now undertake them with the same degree of confidence and assurance that they would undertake to do appendectomies or gall bladder operations.

In any field of surgery, insuperable difficulties will sometimes be encountered, and in the selection of our surgical risks we must be able to estimate those difficulties and thus avoid unnecessary high mortality. It must be remembered that some goiter cases have as late sequelas complications of the heart and kidneys, which place them clear without the possibility of surgical relief. Others of less severe complications may be treated by ligation of the blood supply, reducing their activity and thus carried along until partial compensation takes place, when it will be possible to do a complete operation. In these cases the keenest discrimination must be exercised.

The differentiation of the different types of goiter from a pathological standpoint is not more difficult than that of tumors of other parts of the body. The pathologist of today can tell just how far the goiter has advanced and estimate the damage done to the nervous and circulatory system. But such knowledge to be of value to the surgeon in a particular case must be known before the tumor becomes available to the pathologist. The surgeon must command such knowledge of inaugural symptoms and the pathology of the living and especially of the symptomatology of diseases referable to the thyroid as to enable him to classify his goiters and to estimate the approximate damage done to the system before he can determine the two main questions in the surgery of the thyroid; namely, the time to operate and the type of operation to do.

From a clinical standpoint, all goiters presenting for operation will be divided into one of the following three classes:

1. Simple goiters; that is, simple adenomatous or colloid goiters.
2. Exophthalmic goiters.
3. Malignant goiters, or malignant tumors of the thyroid gland.

The simple goiters are those glands in which the intercellular spaces are filled with colloid material, on which the functionating part of the gland is displaced with a diffuse encapsulated adenomatous growth. In other words, they are the types of goiters which do not oversecrete. It is practical to include as simple goiters those which were formerly hypersecretive and have become passive, also a few of those which were exophthalmic in type and which have reverted back to simple goiters.

The surgical indication for the relief of simple goiters is the enucleation of the diseased or tumor part, following a technic which will leave intact the functionating part of the gland and produce the minimum amount of trauma to the contiguous tissues.

The exophthalmic goiters are characterized by increase in the stroma and parenchyma of the gland, and as a consequence are functionating beyond the limits of normal health, thus producing a chain of symptoms which are grouped under the general term hyperthyroidism, and of which the protruding eye or exophthalmus is the late manifestations or syndrome.

The term exophthalmic goiter should be dropped, as it is really not descriptive of the real disease, but only of one of its very late symptoms. If we wait for the protruding eye in our goiter cases, we will often wait until our patient cannot be cured of her or his trouble.

The special symptoms of hyperthyroidism are referable to the heart and nervous system. The heart symptoms are tachycardia in the early stages, and dilatation and fatty degeneration in the advanced stages. The nervous symptoms are extreme nervousness, irritability, tremors, mental depression, and sometimes mania.

The general symptoms are emaciation, loss of strength, anemia, digestive disturbances, and periodic fevers.

A study of the mortality in goiter operations will show that upon simple colloid or adenomata involve but little risk. If a careful technic is obtained, the death rate should be limited to the unavoidable accidents of surgery.

Patients with those types of goiter will seek operation for relief of symptoms arising from pressure, neuralgia or for esthetic purposes, or relief from the deformity.

The fact that such goiters may be harmless and not productive of symptoms should not deter us from operating on them, for one great important fact should always be given prominence in considering these cases—that is the tendency of all such goiters to malignant degeneration. This fact should be impressed upon every patient. Just what per cent of goiters will become malignant if the patient lives to be sixty years or over is not known, but we believe that it is greater than generally supposed. No specific treatment can be directed against the prevention of malignancy; the only safe precaution is the timely extirpation or enucleation. When symptoms of malignancy become apparent, the case has often progressed beyond the stage of operability, and for that reason, especially considering the rapid and oftentimes sudden development of malignancy during the so-called cancerous age, physicians should warn every goiter patient who reaches the age of forty to be on the alert to observe an increase in size or shape of tumor and for any pains or pressure symptoms and for any lymphatic glandular enlargement about the region of the goiter, and if such symptoms are observed immediate operation should be advised. If the case is operated before the cancer escapes from the capsule and before metastases take place, it may and often will prove successful.

When we come to deal surgically with exophthalmic goiters, we are assuming grave risk in practically every case. The surgery of exophthalmic goiters is difficult, and calls forth the keenest discrimination both as to the time and technic of the operation. As said, the chief indication for operation must be based upon what appears in each individual case to be the safest and surest method of reducing the secretion within the bounds of normal health.

We must remember that we are dealing with a gland which normally has an important function to perform, but which, owing to some stimulus, the nature of which we do not know, has become structurally changed, hypertrophied, and hyperactive. The real danger from the operation of exophthalmic goiter is from an acute exacerbation of symptoms due to absorption of excess secretion. If we can eliminate the one great danger of acute hyperthyroidism, we will be able to reduce operative mortality at least 50 per cent. So far, no writer has assigned an adequate cause for such acute exacerbations following the removal of the gland, which in theory harmonizes with the generally accepted ideas of auto-toxemias. We believe the cause of the post-operative hyperthyroidism to be due to a combination of several conditions, a part of which may be obviated.

The following factors undoubtedly influence and materially aid in causing acute hyperthyroidism following operation, and from observation and deductive reasoning they are of greatest potency in the order named: (1) Trauma, (2) hemorrhage, (3) shock, (4) prolonged anesthesia, and (5) prolonged operation. No technic is adequate which does not minimize these five factors.

All surgeons know of the evil effects of operative trauma, and all that applies in other fields is apparent in trauma following goiter operations, with the additional danger of increased absorption of the secretion which is squeezed from the gland by rough handling. Blood escaping from the gland should not be allowed to flow over the tissue which has separated, and as the operation proceeds salt solution packs should be advanced with the line of separation. As few instruments should be used as possible, and all large vessels should be ligated at once. Every tissue should be handled as gently and as little as possible. The gland itself should be handled with the greatest care. As little hemorrhage as possible should be had. Our first efforts should be directed towards ligation of the superior vessels, and all large arteries and veins should be ligated or cut between forceps. The patient should be placed in position with head elevated about 60 degrees. Shock should be obviated in every way. Of course, trauma, hemorrhages, prolonged anesthesia and operation all tend to produce shock, but other things, if obviated, would tend to minimize it; namely, correct preparation of patient, which means a period of rest, regulation of the other reciprocal organs, etc.

It is needless to discuss the dangers of a prolonged anesthesia and operation. We believe that both are causative agents in producing post-operative hyperthyroidism and are attended with the same danger as apply in other surgical procedures.

---

For premedical, medical and hospital work, a student must expect to give up seven of the best years of his life, with large outlay for room and board, clothing, books and instruments. In addition, the cost of instruction runs from $350 to $2000 in class A schools.—*C. R. Bardeen, Wisconsin Medical Journal.*

